# Intensity, frequency, duration, and volume of physical activity and its association with risk of depression in middle- and older-aged Chinese: Evidence from the China Health and Retirement Longitudinal Study, 2015

**DOI:** 10.1371/journal.pone.0221430

**Published:** 2019-08-19

**Authors:** Ruoxi Wang, Ghose Bishwajit, Yongjie Zhou, Xiang Wu, Da Feng, Shangfeng Tang, Zhuo Chen, Ian Shaw, Tailai Wu, Hongxun Song, Qian Fu, Zhanchun Feng

**Affiliations:** 1 School of Medicine and Health Management, Tongji Medical College, Huazhong University of Science and Technology, Wuhan, Hubei, China; 2 School of International Development and Global Studies, University of Ottawa, Ottawa, Canada; 3 Research Center for Psychological and Health Sciences, China University of Geosciences, Wuhan, China; 4 Affiliated Mental Health Center of Tongji Medical College, Huazhong University of Science and Technology, Wuhan, Hubei, China; 5 School of Pharmacy, Tongji Medical College, Huazhong University of Science and Technology, Wuhan, Hubei, China; 6 College of Public Health, University of Georgia, Athens, GA, United States of America; 7 School of Sociology and Social Policy, University of Nottingham, Nottingham, United Kingdom; Indiana University-Purdue University Indianapolis, UNITED STATES

## Abstract

**Background:**

The general benefit of physical activity (PA) to one’s mental health has been widely acknowledged. Nevertheless, the specific type and amount of PA that associates with lower risk of depression in China awaits further investigation. The present study was conducted on middle- and older-aged Chinese population with two objectives: 1) to understand the patterns of PA; 2) to measure the associations between depression and PA at different levels from various aspects.

**Methods:**

Using data from the China Health and Retirement Longitudinal Study (CHARLS, 2015), we selected 9118 community residents aged 45 years and older. Depressive symptoms were measured by 10-item Center for Epidemiologic Studies (CES-D 10). Multivariate logistic regression model was performed to examine the association between risk of depression and PA from four aspects including intensity, frequency, duration, and volume.

**Results:**

Spending 1–2 days/week (OR = 0.58, 95% CI: 0.36, 0.91), less than 30 minutes each time (OR = 0.66, 95% CI: 0.42, 1.03) or 150–299 min/week (OR = 0.49, 95% CI: 0.28, 0.87) on Moderate Physical Activity (MPA) was associated with lower odds of depression in women. Spending 3–5 days/week (OR = 1.98, 95% CI: 1.29, 3.05) or 6–7 days/week (OR = 1.50, 95% CI: 1.07, 2.11), 4 hours and longer each time (OR = 1.65, 95% CI: 1.18, 2.32), 300 min/week or longer (OR = 1.65, 95% CI: 1.22, 2.24) on Vigorous Physical Activity (VPA) in total, or 2250 Metabolic Equivalent of Task (OR = 1.73, 95% CI: 1.26, 2.38) on Moderate-to-Vigorous PA was associated with higher risk of depression in men.

**Conclusions:**

The association between depression and PA depended largely on intensity and gender. Lower frequency, shorter duration, and moderate amount of MPA was associated with lower risk of depression in women. Risk of depression was higher in men who spent higher frequency, longer duration, and overlong time on VPA.

## Introduction

Depression, one type of non-communicable diseases, has triggered great public health concerns in both developed and developing countries due to its high prevalence and heavy burden of diseases [1]: it is one of the most prevalent disorders [2], affects nearly 350 million people, and accounts for 12.7% of all-cause mortality around the globe [3]. Moreover, it leads to other health problems such as type 2 diabetes [4], cardiovascular diseases [5], and suicide [6]. These secondary comorbidities further affirm the tremendous burden that depression accounts for. China, home for nearly 18% of the global population, encounters a great threat posed by depression: with nearly 17% of the global disease burden of psychiatric-related illness [7], of which 30% can be attributed to depression. The figure is even more threatening in working age adults: 43% of mental disorder related disease adjusted life years (DALYs) [8]. Considering both the pattern that depressive symptomology increases as one ages [9, 10] and the current trend of population ageing in China [11], the threat will possibly be enlarged.

Conventional interventions such as cognitive behavioral therapy and antidepressants have been recognized as effective means for depression treatment, nevertheless, many are confined to a small scale of population due to their high demands for professional resources [12]. Similar to other low and middle income countries (LMICs), China also suffers from a profound gap between need and supply in mental health services [13]. This suggests that the above-mentioned approaches can hardly be an effective approach to solve the large-scale problem facing China nor other LMICs [12], and therefore, novel approaches with greater accessibility are urgently needed.

In recent years, emerging evidence started to regard Physical Activity (PA) as an important solution, considering its low demand for professional resources and contribution to one’s physical health (such as health function and sleep duration) and emotional social support [14]), which are closely related to one’s depressive symptoms [15]. In this case, various organizations have published recommendations on PA. For instance, WHO suggests older adults taking at least 75 minutes of Vigorous Physical Activity (VPA) or at least 150 minutes of Moderate Physical Activity (MPA) or an equivalent combination of moderate-to-vigorous PA (MVPA) each week [16]; The American Heart Association further encourages the elderly to spend at least 30 minutes per day on 5 days of the week on MPA or at least 20 minutes on 3 days of the week on VPA [17]. However, the exact role of PA on depression awaits statistical validation.

Among the limited number of studies that attempted to investigate the protective role of PA on depression, findings turned out to be largely heterogeneous. Some studies generally concluded with PA’s protective effect on depression [18, 19]; other studies have further investigated PA from various dimensions, including intensity, duration and volume, and resulted in more complex findings: some indicated that individuals who engaged in VPA/MPA [20–22] or PA with higher frequency [23, 24] had lower odds of depression; nevertheless, some suggested that LPA rather than VPA/MPA [25–27], lower frequency (1–2 times/week) [28], or PA with smaller volume than recommended [29–31] was protective. Moreover, some indicated that the association differed significantly with gender [29, 32]. One study concluded with no statistically significant associations between depression and PA of any kind [28], and one even found that people who engaged in high volume of PA were more likely to suffer from depression [33]. The lack of consensus regarding this issue on the one hand indicates the association between PA and depression depends largely on the specific cultural and demographic contexts [34], and on the other hand, suggests that PA is a multidimensional construct that consists of intensity, frequency, duration and volume, and the relationship cannot be fully comprehended by a single dimensional analysis [35].

Moreover, Chinese citizens are greatly influenced by Eastern culture and present distinctive patterns of PA: East Asians tend to take fewer physical activities and at lower intensity than those in Western countries [30]. Thus, whether the PA recommendations that have been designed and subject to Western population fit Eastern context and how PA is associated with better mental health in middle- and older-aged Chinese residents still awaits further investigation. To the best of our knowledge, no study has examined the correlation between depression and PA in China by taking into consideration all four key dimensions of PA (intensity, frequency, duration and volume). To this regard, we conducted this study on middle- and older-aged Chinese with two objectives: 1) to understand the patterns of PA; 2) to measure the associations between depression and PA from various aspects including intensity, frequency, duration and volume.

## Materials and methods

### Study sample

We extracted our data source from the China Health and Retirement Longitudinal Study (CHARLS 2015) Wave 4 conducted in 2015. CHARLS is a nationwide survey program covering 450 villages and 150 counties in 28 provinces with an aim to provide comprehensive and quality data on the demographic background, family characteristics, health status, work, and retirement status of the mid-aged and older residents in China. The data were collected using a four-stage, stratified, cluster sampling method. Detailed sampling technique was documented in Zhao et al.’s study [36]. The CHALRS research team has obtained ethical approval from the institutional review board (IRB) at Peking University. Amongst 20,967 surveyed community residents, 9,118 participants were selected for this cross-sectional study according to the following criteria: 1) aged 45 and older, 2) gave information on whether or not they had conducted any type of PA (VPA/MPA/LPA), 3) were assigned a case weight to balance the sample with the source population.

### Variables

#### Outcome variables

Depression: a 10-item Center for Epidemiology Studies Depression Scale (CES-D 10) was used to screen depression. The answers for CES-D 10 are on a four-scale metrics coding from 0 to 3. The total score ranges from 0 to 30, with higher scores indicating more depressive symptoms. The CES-D 10 has been used in previous studies and showed good reliability (Cronbach’s alpha = 0.815) [37]. Several studies have reported a cut-off point of 12 with good validity to identify clinically significant depression [38, 39]. In this case, participants with a CES-D 10 score of 12 or above were regarded as in risk of depression.

#### Main explanatory variables

CHARLS classified PA into 3 levels according to intensity: 1) VPA: activities that require hard/high intensity physical effort and make one breathe much harder than normal (e.g. heavy lifting, digging, aerobics, fast bicycling, cycling with a heavy load, etc.); 2) MPA: activities that can result in breathing somewhat harder than normal (e.g. carrying light loads, bicycling at a regular pace, mopping the floor); 3) LPA: walking, including walking at work, travelling from place to place, and walking for recreation.

Participants were asked: 1) did they conduct VPA/MPA/LPA for at least 10 minutes continuously for a usual week; 2) if yes, how many days they normally took VPA/MPA/LPA in a week; and 3) how much time (<30min, <2h, <4h, ≥4h) they spent on PA each time.

Frequency: answers for VPA/MPA/LPA ranged from 0-7days/week, and were categorized into never (0 days)/ 1–2 days/week/ 3-5days/week/ 6-7days/week.

Duration: this study took into consideration that some participants did not took any PA, and therefore, categorized this variable into 5 scales, including no PA/<30 min per time / 30–119 min per time / 120–239 min per time / ≥240 min per time.

Volume: According to the WHO international guideline [16] for PA, at least 75min/week VPA, or 150min/week MPA, or 150min/week MVPA was recommended for the elderly. In addition, WHO has also warned that VPA/MPA over 300min/week may be harmful to the senior population. In this case, we calculated the total length of VPA/MPA/LPA by: 1) estimating the daily duration of VPA/ MPA/ LPA using the average value of each time; 2) using the following formula:

VolumeofVPA/MPA/LPA=FrequencyofVPA/MPA/LPA*DurationofVPA/MPA/LPA

Regarding volume of VPA, responses were classified into 4 groups: no VPA/ <75 min/week <300 min/week/ ≤300 min/week; whereas regarding total length of MPA, responses were classified into 4 groups: no MPA/ <150 min/week/ <300 min/week/ ≥300 min/week. Due to the lack of international guidelines for LPA, we adjusted total length of LPA by 5 levels: no LPA/ 1^st^ quartile (≤105 min/week)/ 2^nd^ quartile (≤525 min/week)/ 3^rd^ quartile (≤1260 min/week)/ 4^th^ quartile (>1260 min/week). In terms of MVPA, The score was computed by multiplying the length of VPA and MPA by an assigned metabolic equivalent value (MET): MPA = 4 MET, VPA = 7.5 MET [26]. Since the lower and higher cut-off points for the sufficient MVPA are 150 min/week MPA and 300 min/week VPA, which equal to 600 MET and 2250 MET, respectively. Therefore, the volume of MVPA was then coded as no MVPA/ not sufficient (<600 MET)/ sufficient (600–2249 MET)/ overlong (≥2250 MET).

#### Control variables

This study included socio-demographic variables, health behaviors and health status related variables for adjustment. Socio-demographic variables included: gender (male, female), age (45–54, 55–64, 65–74, ≥75), residency (urban, rural), education (illiterate, primary school and lower, middle school, high school and higher), marital status (married/cohabitating, single), living near children (no/ yes), total annual household income (1^st^ quartile (≤$232/y), 2^nd^ quartile (≤$1279/y), 3^rd^ quartile (≤$5338/y), 4^th^ quartile (>$5338/y)). Health behavior-related variables included: alcohol intake (no, yes), and smoking (no, yes). Health status-related variables included: BMI (underweight (<18.5 kg/m^2^), normal (18.5–24.99 kg/m^2^), overweight (25–29.99 kg/m^2^), obese (≥30 kg/m^2^)), diabetes (no, yes), and hypertension (no, yes).

### Data analysis

Frequencies and percentages were calculated for descriptive data. Associations between levels of PA and outcome variables were assessed by binary logistic regression with the potentially confounding variables being adjusted. Adjusted Odd Ratios (ORs) and their 95% Confidence Intervals (95% CIs) were presented as measures of effect. Case weights (provided by the CHARLS study) were used to adjust for the stratified sampling method and non-response patterns. Data were analyzed using R Version 3.5.1.

## Results

### Characteristics of the participants

Basic characteristics of the whole sample population, as well as of those who were with or without risk of depression are shown in Table 1. Of the 9,118 participants, a greater proportion were female, in the age group of 45–74 years, rural residents, with a primary school or lower level of education, partnered and living near children. The majority of respondents did not consume alcohol or smoke; have not been diagnosed as diabetes or hypertension; and were either in normal weight or overweight.

**Table 1:** Sample characteristics of middle- and older-aged Chinese residents.

	Total		No Risk (n = 6823)		Risk (n = 2295)	
	n^a^	%^b^	n^a^	%^b^	n^a^	%^b^
**Gender**						
Male	4437	49.02	3635	53.32	802	34.55
Female	4681	50.98	3188	46.68	1493	65.45
**Age**						
45–54	3176	35.06	2483	36.35	693	30.75
55–64	3168	33.89	2358	33.74	810	34.39
65–74	2002	21.53	1422	20.51	580	24.94
≥75	772	9.52	560	9.40	212	9.92
**Residency/Hukou**						
Urban	3589	48.72	2877	52.09	712	37.42
Rural	5529	51.28	3946	47.91	1583	62.58
**Education**						
illiterate	2284	22.81	1476	19.78	808	32.99
≤primary school	4024	43.12	2997	42.79	1027	44.22
middle school	1767	19.91	1422	20.89	345	16.62
≥high school	1033	14.16	921	16.54	112	6.18
**Marital status**						
divorced/widowed/single	1115	12.86	702	11.21	413	18.38
married/partnered	8003	87.14	6121	88.79	1882	81.62
**Living near children**						
No	1254	14.32	938	14.55	316	13.52
Yes	7618	85.68	5716	85.45	1902	86.48
**Total household income**						
1^st^ quartile (lowest)	1078	23.31	718	21.19	360	29.60
2^nd^ quartile	1034	22.04	698	19.86	336	28.48
3^rd^ quartile	1066	24.00	800	24.05	266	23.82
4^th^ quartile (highest)	1052	30.65	886	34.89	166	18.10
**Alcohol intake**						
No	5855	63.35	4204	60.70	1651	72.25
Yes	3257	36.65	2613	39.30	644	27.75
**Smoking**						
No	6504	71.70	4747	70.28	1757	76.46
Yes	2609	28.30	2073	29.72	536	23.54
**Diabetes**						
No	6930	88.40	5191	89.40	1739	85.23
Yes	861	11.60	574	10.60	287	14.77
**Hypertension**						
No	5099	63.80	3894	65.57	1205	58.23
Yes	2789	36.20	1939	34.43	850	41.77
**BMI level**						
Under weight	433	5.77	297	5.35	136	7.11
Normal Weight	4289	57.84	3152	56.86	1137	60.95
Overweight	2265	31.30	1754	32.65	511	26.97
Obese	396	5.09	296	5.13	100	4.98

N.B.

^a^: unweighted frequency

^b^: weighted column percentage. To some variables, the total percentage may not equal to 100 due to rounding.

According to CES-D 10 scale, over one fourth of the participants were in risk of depression. Generally speaking, participants with depression were more likely to be female, aged 65 and older, in rural areas, with a primary school or lower level of education, with no partner, with lower family income (first two quartiles), non-drinker, non-smoker, with diabetes or hypertension, and in normal weight or underweight.

### Frequency of PA in men and women

**Fig 1:**
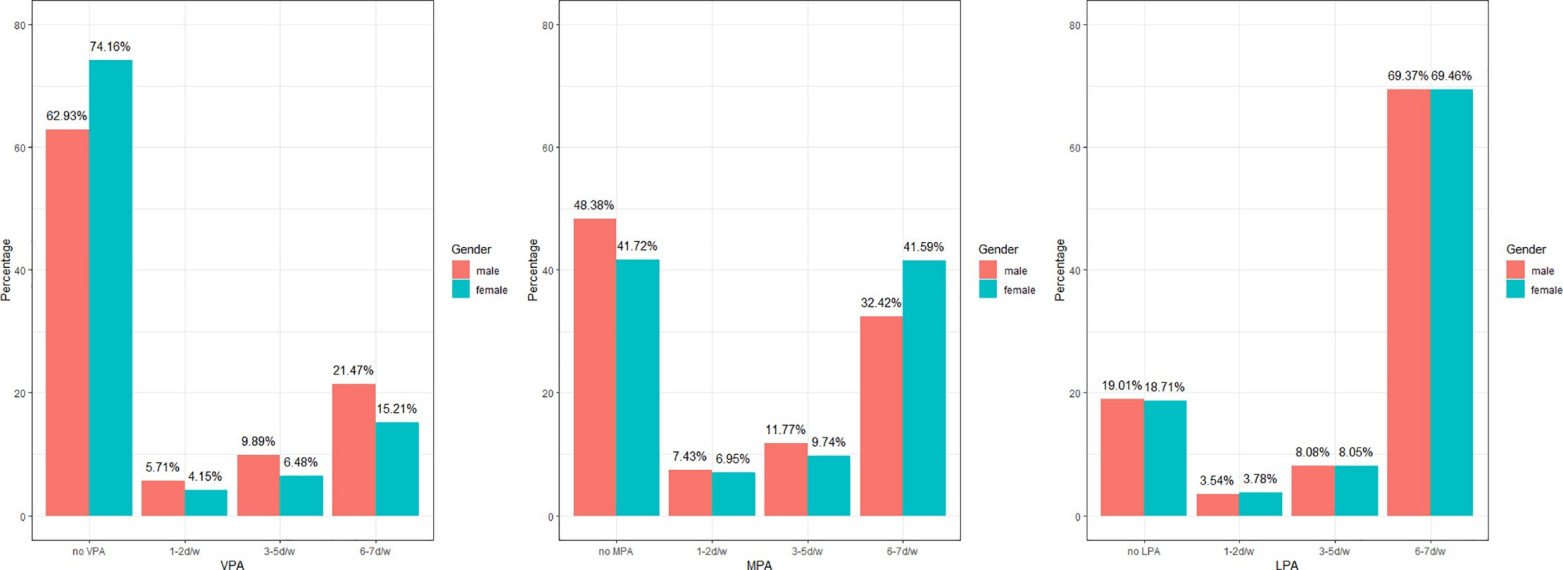
Frequency of VPA, MPA and LPA in men and women.

Generally speaking, the proportion of respondents taking PA increased as the PA intensity decreased in both men and women (31.30% for VPA, 55.04% for MPA and 81.14% for LPA). A phenomenon that the majority of participants either took no PA or had frequent PA (6-7days/week) was observed in all three levels PA. Despite that in LPA, the distribution differed between men and women in VPA and MPA: A larger proportion of men taking part in VPA whereas a significantly larger proportion of women involved in MPA, especially in frequent MPA (Fig 1).

### Duration of PA in men and women

**Fig 2:**
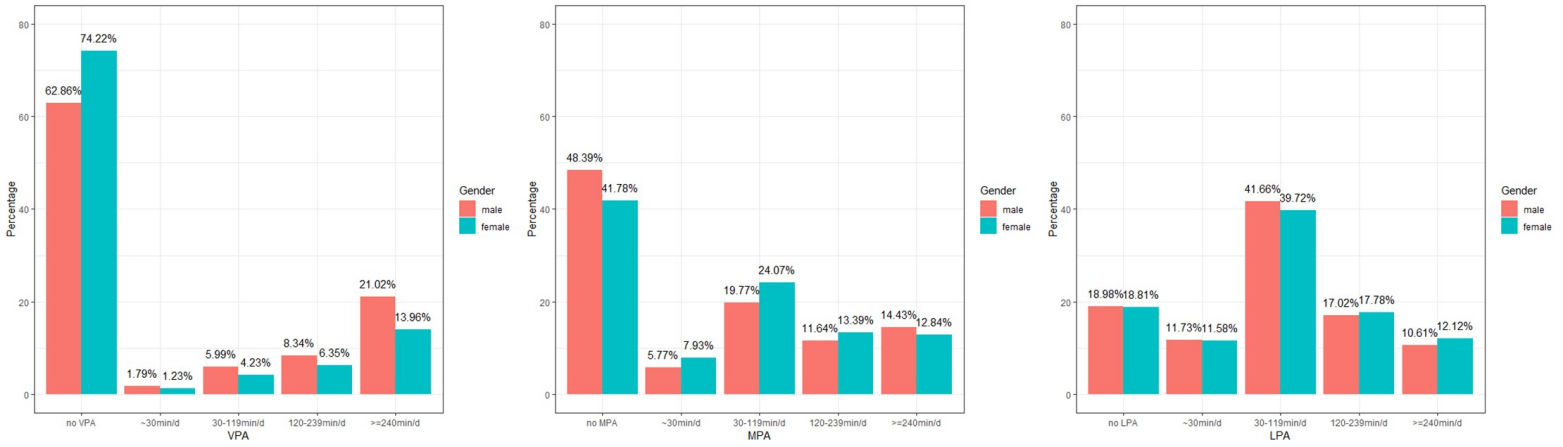
Duration per time for VPA, MPA and LPA in men and women.

Amongst those who took VPA, the majority of men and women spent more than 4 hours per day. Different patterns between men and women were observed in MPA duration: amongst those who took MPA, a significantly larger proportion of women spent 30–119 minutes on MPA each time, whereas the distribution was more evenly in men. Women and men shared similar patterns regarding LPA duration (Fig 2).

### Volume of PA in men and women

**Fig 3:**
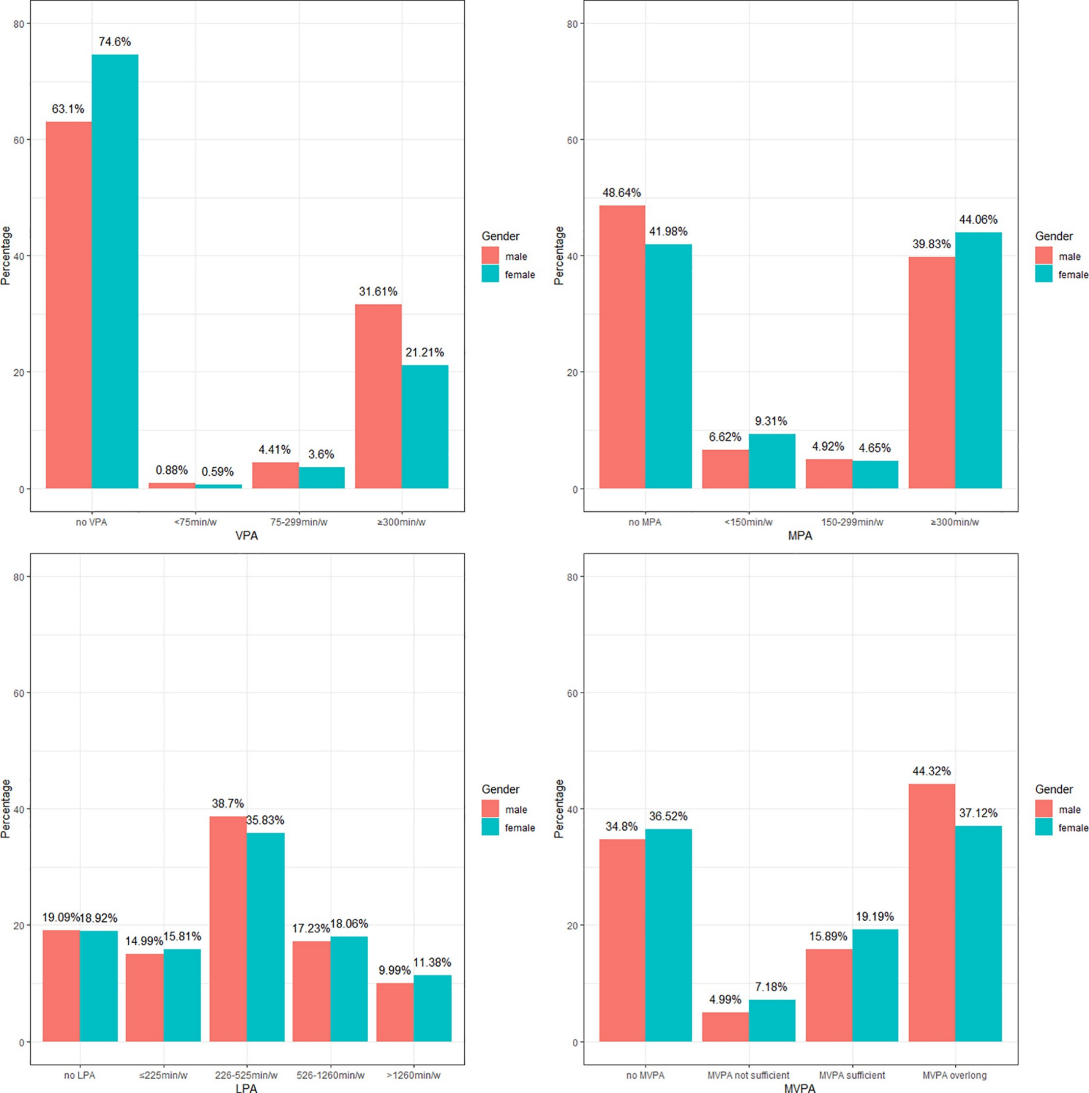
Volume of VPA, MPA, LPA and MVPA per week in men and women.

Fig 3 summaries the total length of time the participants spent on VPA, MPA, LPA and MVPA each week. Similar to Fig 1, the distribution was heavily bipolarized in VPA, MPA and total MVPA: there was a large proportion of participants who did not take any PA, whereas at the same time, a considerably large proportion spent much more time on PA than they were recommended to. A significantly larger proportion of men spent over 300 min/week on VPA whereas a significantly larger proportion of women spent over 300 min/week on MPA. In total, over 58% men and women took sufficient amount of MVPA as they were recommended to.

### Relationship between PA and depression in men and women

Table 2 outlines the associations between different dimensions (intensity, frequency, duration and volume) of PA and risk of depression in the whole sample population, and compares the differences between men and women.

**Table 2:** Associations between depressive risk and PA frequency, duration and volume.

	Model 1: Whole sample		Model 2: Female		Model 3: Male	
	OR	95%CI	OR	95%CI	OR	95%CI
**FREQUENCY**						
**VPA**						
No activity	1		1		1	
1-2d/w	1.04	0.71, 1.50	0.93	0.56, 1.55	1.19	0.68, 2.07
3-5d/w	**1.57**w	**1.18, 2.08**	1.25	0.84, 1.85	1.98**	**1.29, 3.05**
6-7d/w	**1.24+**	**0.99, 1.54**	1.07	0.80, 1.43	1.50*	**1.07, 2.11**
**MPA**						
No activity	1		1		1	
1-2d/w	0.89	0.64, 1.25	0.58*	**0.36, 0.91**	1.57	0.95, 2.59
3-5d/w	0.86	0.64, 1.14	0.82	0.57, 1.19	0.91	0.57, 1.47
6-7d/w	1.13	0.93, 1.37	0.97	0.76, 1.24	1.45*	**1.05, 2.00**
**LPA**						
No activity	1		1		1	
1-2d/w	0.74	0.47, 1.18	0.74	0.42, 1.30	0.78	0.34, 1.79
3-5d/w	1.11	0.79, 1.56	1.09	0.70, 1.70	1.14	0.67, 1.94
6-7d/w	0.90	0.73, 1.11	0.92	0.72, 1.20	0.86	0.61, 1.23
**DURATION PER TIME**						
**VPA**						
No activity	1		1		1	
<30min	1.54	0.81, 2.92	1.36	0.56, 3.27	1.53	0.58, 4.06
30-119min	0.91	0.61, 1.34	0.78	0.46, 1.32	1.18	0.66, 2.11
120-239min	1.07	0.78, 1.47	0.90	0.60, 1.36	1.40	0.86, 2.29
≥240min	1.39**	**1.12, 1.74**	1.24	0.91, 1.64	1.65**	**1.18, 2.32**
**MPA**						
No activity	1		1		1	
<30min	0.80	0.55, 1.16	**0.66+**	**0.42, 1.03**	1.22	0.63, 2.39
30-119min	1.12	0.88, 1.41	0.92	0.69, 1.23	1.57*	**1.06, 2.33**
120-239min	0.99	0.76, 1.28	0.82	0.59, 1.14	1.31	0.86, 1.99
≥240min	1.07	0.82, 1.38	1.06	0.76, 1.48	1.05	0.69, 1.59
**LPA**						
No activity	1		1		1	
<30min	0.89	0.66, 1.22	1.03	0.7,1 0.51	0.65	0.37, 1.12
30-119min	0.86	0.68, 1.08	0.85	0.64, 1.14	0.86	0.58, 1.28
120-239min	1.07	0.82, 1.39	0.98	0.70, 1.38	1.21	0.78, 1.86
≥240min	0.95	0.70, 1.28	0.96	0.66, 1.40	0.95	0.58, 1.56
**VOLUME**						
**VPA**						
No activity	1		1		1	
<75min/w	1.59	0.64, 3.97	1.57	0.43, 5.79	1.33	0.35, 5.14
<300min/w	0.92	0.60, 1.4	1.00	0.59, 1.70	0.81	0.39, 1.67
≥300min/w	1.31**	**1.08, 1.59**	1.09	0.84, 1.42	1.65**	**1.22, 2.24**
**MPA**						
No activity	1		1		1	
<150min/w	0.89	0.63, 1.26	0.73	0.48, 1.11	1.35	0.74, 2.48
150-299min/w	**0.65+**	**0.41, 1.02**	0.49*	**0.28, 0.87**	0.96	0.46, 2.01
≥300min/w	1.10	0.91, 1.33	0.96	0.75, 1.22	**1.33+**	**0.98, 1.81**
**LPA**						
No activity	1		1		1	
≤105min/w	0.88	0.67, 1.17	0.96	0.68, 1.37	0.74	0.45, 1.21
≤525min/w	0.86	0.68, 1.09	0.84	0.62, 1.13	0.90	0.60, 1.34
≤1260min/w	1.08	0.83, 1.41	1.04	0.74, 1.45	1.19	0.78, 1.84
>1260min/w	0.91	0.67, 1.23	0.95	0.65, 1.40	0.87	0.53, 1.45
**MVPA**						
No activity	1		1		1	
<600 MET	0.93	0.62, 1.38	0.81	0.50, 1.31	1.15	0.56, 2.37
600–2249 MET	1.02	0.78, 1.33	0.90	0.66, 1.23	1.22	0.75, 200
≥2250 MET	1.22*	**1.01, 1.47**	0.96	0.75, 1.23	1.73***	**1.26, 2.38**

N.B. ORs were adjusted for age, gender, residency, education, marital status, living near children, total household income, alcohol intake, smoking, diabetes, hypertension, BMI level and case weights

+: p<0.1

*: p<0.05

**: p<0.01

***: p<0.001.

Regarding frequency, the participants who spent 3 days or more on VPA had significantly higher risk of depression (OR = 1.57, 95% CI: 1.18, 2.08 for 3–5 days/week and OR = 1.24, 95% CI: 0.99, 1.54 for 6–7 days/week, respectively) than those who did not take VPA. The association was statistically significant in men but not in women. However, spending 1–2 days/week on MPA was associated with lower risk of depression in women (OR = 0.58, 95% CI: 0.36, 0.91), whereas spending 6–7 days/week was associated with 1.45 times higher likelihood (95% CI: 1.05, 2.00) of depression in men. No significant association was observed between any level of LPA frequency and depression in the whole sample population or subgroups.

Regarding duration, spending over 4 hours on VPA each time was correlated with greater odds of depression (OR = 1.39, 95% CI: 1.12, 1.74). The association was strong in men (OR = 1.65, 95%CI: 1.18, 2.32) but not statistically significant in women (OR = 1.24, 95% CI: 0.91, 1.64). Spending less than 30 min/day on MPA was associated with better mental health status in women, however, men who took 30–119 min/day MPA had 1.57 times higher risk (95% CI: 1.06, 2.33) of depression compared to those who reported not being involved in MPA. No significant association between any level of LPA duration and risk of depression in the whole sample population or subgroups was observed.

Regarding volume, spending more than 300 min/week on VPA was associated with greater odds of depression (OR = 1.31, 95% CI: 1.08, 1.59) in the whole sample and in men (OR = 1.65, 95% CI: 1.22, 2.24). Similar patterns were observed in MVPA. Taking 150–299 min/week MPA was associated with smaller odds of depression in the whole sample population (OR = 0.65, 95% CI: 0.41, 1.02) and in women (OR = 0.49, 95% CI: 0.28, 0.87). Nevertheless, men taking 300 min/week or more on MPA was associated with higher likelihood (OR = 1.33, 95% CI: 0.98, 1.81) of depression.

## Discussion

To the best of our knowledge, the study is the first one to examine the risk of depression in relation to the intensity, frequency, duration, and volume of PA in community-dwelling Chinese adults using a nationwide sample, and also, one of the very few studies to depict the patterns of PA in middle- and older-aged Chinese adults.

### Prevalence of depression

In summary, the findings revealed high prevalence of risk of depression in middle- and older- aged Chinese adults (25.17%) and an upward trend (from 21.82% to 27.46%) with increasing age level. This finding is in agreement with those from recent studies, indicating that 23.6% to 27.0% of the elderly Chinese confront depression, while the risk of depression rises as age increases [40, 41]. The figure is significantly higher than that was estimated two decades ago (3.86%) [42]. Moreover, it is much higher than the average level of LMICs (1%) [43, 44] and even some developed countries [25, 45]. This, on the one hand, indicates the tremendous threat facing China in terms of the rising disease burden of depression; and on the other hand, reiterates the urgent need to identify approaches with broad accessibility to address this issue in a context where professional resources are scarce. Meanwhile, the present study found gender difference on the prevalence of depressive risk: the prevalence in women was significantly greater than that in men. Similar findings can be retrieved from other studies against Chinese older adults [46, 47]. This indicates that older women are in higher need for effective approaches to prevent depression.

### Patterns of PA

Generally speaking, around three fifths of the participants reached the threshold outlined in the WHO guidelines. This figure is similar to those reported in other studies subject to older Chinese adults [48] or elderly in LMICs [32], and supports the statements Wen et al. [31] and Chang et al. [30] made that East Asians tend to engage in PA at lower intensity.

Significant differences in the frequency, duration and volume of PA were observed between different levels of PA intensity and different genders. Generally speaking, men were more active in VPA and women were more active in MPA. Significantly uneven distribution of frequency and volume was observed in VPA and MPA. These findings are consistent with those extracted from previous studies [26, 28, 35]. The reasons may be attributed to the purpose of PA: most of the elderly who take MVPA are for domestic or occupational purposes [49, 50], therefore, tend to have a high frequency and volume of PA. The patterns of duration can serve as indirect evidence for the above speculation: the majority of those who engaged in VPA spent more than 4 hours each time, which often occurs when one engages in a job that requires heavy labor work. In contrast, the distribution of MPA duration was more dispersed between “30-119min per time”, “120-239min per time” and “>240min per time”, whereas a relatively larger proportion of respondents reported 30–119 minutes each time. Taking into consideration that the prevalence of MPA was significantly higher in women than that in men, we speculate that respondents who engaged in MPA were mainly for domestic purpose.

### Associations between PA and depression

Generally speaking, risk of depression was correlated with the intensity, frequency, duration and volume of PA, whereas the direction and type of correlation mainly depended on the intensity and gender.

Regarding VPA, compared with those who took no VPA, those who had higher frequency (3–5 days/week or 6–7 days/week), longer duration (≥240 min/day) and greater length (≥300 min/week) had greater odds of suffering from depression. However, the statistically significant correlation was only found in male but not in women. This may be due to the purpose of VPA: on the one hand, in traditional Chinese culture, men are the ones who take major responsibility to support family [51, 52]; on the other hand, the higher frequency, longer duration and larger volume of heavy-labor work may indicate lower household income and heavier financial burden. This, therefore, may lead to men suffering from higher risk of depression. Regarding adverse effect, similar findings have been documented in some studies [33, 53], and the finding complies with WHO guidelines that indicate the potential harm MVPA may pose on one’s health when the total length exceeds 300 min/week [16]. Strategies such as encouraging lower frequency and smaller amount but not large volume of VPA, or promoting taking PA with happiness may be worth consideration.

Regarding MPA, engaging in sufficient but less than 300min/week MPA was associated with lower risk of depression for the whole sample population, whereas different magnitudes were observed between women and men. Compared with those who did not have MPA, women who had smaller frequency (1–2 days/week), shorter duration (<30 min/day) and moderate length (150–299 min/week) of MPA were in lower risk of depression. However, this inverse associated was not observed in men. Moreover, engaging in higher frequency (6–7 days/week) and greater length (≥300 min/week) of MPA was positively correlated with higher likelihood of depression in men. Similar findings can be retrieved from several recent studies that found PA, especially MPA, more effective in terms of reducing risk of depression in women than men [32, 54]. Some even reported that only women, but not men, benefited from taking part in PA [29, 55]. The potential reason for this gender difference may be attributed to women’s more developed connectedness [56] and their better ability to benefit from the social aspects of PA than men [29]. Our speculation can be underpinned by one study, in which PA was associated with depression but the associated become statistically insignificant when social network was considered [57]. These findings reveal the necessity to consider the gender difference when developing intervention strategies. In addition, we found that the threshold for a statistic significance was lower than that was recommended in some western guidelines [16, 17]. This indicates that the condition varies between different populations and intervention strategy design should be rooted in the specific context. For instance, in light of large proportion of no MPA and small proportion 1–2 day/week MPA in women, interventions could be designed to promote moderate volume of regular MPA. Some experience could be extracted from British policy that encourages MPA such as housework, gardening and home maintenance tasks [58].

Regarding LPA, no statistically significant association was observed between risk of depression and LPA of any kind. This finding can be supported by previous literature that found no significant association between walking and depression [58] and it can be further underpinned by WHO guidelines in which recommendations have only been given to VPA and MPA but not to LPA [16]. However, we noted that some studies reported benefits LPA brought to one’s risk of depression [29]. The difference in outcome may be attributed to two reasons: first, walking was part of everyday life to most of the Chinese residents and its contribution to one’s physical health is limited compared to moderate or intense PA [27, 58]. Second, as one study further divided LPA into high-light PA and low-light PA, and found high-light PA but not low-light PA was related to better well-being [27], the insignificant associated found in this study may be due to the unavailability to further divide LPA.

### Strength and limitations

To the best of our knowledge, this is the first study to explore the association between risk of depression and PA from multiple dimensions including intensity, frequency, duration and volume in middle- and older-aged Chinese using a nationwide dataset. Additionally, this study is one of the few studies that further investigate the correlation by taking gender difference and four key dimensions of PA into consideration. Finally, as Rothon et al [59] indicated, one of the weaknesses of the prior studies is the failure to control for other clinically confounding factors. Health behaviors (such as smoking and drinking) and health status (diabetes, hypertension and BMI) related variables were considered and adjusted in this study.

The findings should be interpreted with caution due to the following limitations. First, although we have observed some associations between physical activity and depression, we are not able to establish a causal relationship between these two variables using a cross-sectional study. Identification of causal relationships will have to await future research using a longitudinal design. Second, PA was self-reported, which may result in recall bias and underestimation of PA. Also, different from the International Physical Activity Questionnaire and Global Physical Activity Questionnaire that ask the exact length of time one spends on PA (numbers of minutes/hours), the questionnaire used in CHARLS only classified the duration into four categories (<30min, <2h, <4h, ≥4h), which may result in lower validity in calculating the total volume of PA. Further studies may benefit from assessing PA using objective measures (such as accelerometers). Finally, the present study was carried out subject to community-dwelling residents, and therefore, may not be generalizable to the institutionalized individuals.

## Conclusions

In conclusion, the findings revealed a considerable high prevalence of risk of depression in middle- and older-aged Chinese, especially in women, which calls for attention and more cost-effective approaches to address this issue. This study found that the risk of depression was correlated with the intensity, frequency, duration, and volume of PA, whereas the direction and magnitude mainly depended on the intensity and gender. To be specific, after controlling one’s demographic, health behaviors and physical health status, men with higher frequency, longer duration and greater length of VPA were associated with higher odds of suffering from depression than those who did not engage in VPA. No such association was found in women. Compared with not taking any MPA, smaller frequency, shorter duration and moderate volume of MPA were associated with lower risk of depression in women. However, similar associations have not been found in men. These findings not only serve to further understand the relationships between PA and depression in middle- and old-aged adults in China, but also remind the need to consider China’s cultural context when designing interventions according to international guidelines. The latter can help to inform policymakers to consider these variations to design target strategies with higher effectiveness.

## Abbreviations

WHO: World Health Organization
CHALRS: China Health and Retirement Longitudinal Survey
PA: physical activity
VPA: vigorous physical activity
MPA: moderate physical activity
LPA: light physical activity
MVPA: moderate-to-vigorous physical activity
LMICs: low- and middle- income countries
CES-D 10: 10-item Center for Epidemiologic Studies
MET: metabolic equivalent of task
YLD: years lived with disability
DALY: disease adjusted life years

## References

[1] Malhi GS, Mann JJ. Depression. Lancet. 2018;392(10161):2299–312. 10.1016/S0140-6736(18)31948-2 WOS:000451067600029. 30396512

[2] Liu QL, Cai H, Yang LH, Xiang YB, Yang G, Li HL, et al Depressive symptoms and their association with social determinants and chronic diseases in middle-aged and elderly Chinese people. Sci Rep. 2018;8 10.1038/s41598-018-22175-2 WOS:000426354200013. 29497126 PMC5832867

[3] Walker ER, McGee RE, Druss BG. Mortality in mental disorders and global disease burden implications: A systematic review and meta-analysis. JAMA Psychiatry. 2015;72(4):334–41. 10.1001/jamapsychiatry.2014.2502 WOS:000352487000005. 25671328 PMC4461039

[4] Vancampfort D, Correll CU, Galling B, Probst M, De Hert M, Ward PB, et al Diabetes mellitus in people with schizophrenia, bipolar disorder and major depressive disorder: a systematic review and large scale meta-analysis. World Psychiatry. 2016;15(2):166–74. 10.1002/wps.20309 WOS:000379302100020. 27265707 PMC4911762

[5] Seldenrijk A, Vogelzangs N, Batelaan NM, Wieman I, van Schaik DJF, Penninx BJWH. Depression, anxiety and 6-year risk of cardiovascular disease. J Psychosom Res. 2015;78(2):123–9. 10.1016/j.jpsychores.2014.10.007 WOS:000348632400004. 25454680

[6] Isacsson G, Rich CL, Jureidini J, Raven M. The increased use of antidepressants has contributed to the worldwide reduction in suicide rates. Br J Psychiatry. 2010;196(6):429–33. 10.1192/bjp.bp.109.076166 WOS:000278427800004. 20513850

[7] Liu S, Page A. Reforming mental health in China and India. Lancet. 2016;388(10042):314–6. 10.1016/S0140-6736(16)30373-7 WOS:000380016900007. 27209145

[8] Charlson FJ, Baxter AJ, Cheng HG, Shidhaye R, Whiteford HA. The burden of mental, neurological, and substance use disorders in China and India: a systematic analysis of community representative epidemiological studies. Lancet. 2016;388(10042):376–89. 10.1016/S0140-6736(16)30590-6 WOS:000380016900041. 27209143

[9] Burns RA, Butterworth P, Luszcz M, Anstey KJ. Stability and change in level of probable depression and depressive symptoms in a sample of middle and older-aged adults. Int Psychogeriatr. 2013;25(2):303–9. 10.1017/S1041610212001470 WOS:000312529100017. 22906419

[10] Beard JR, Bloom DE. Towards a comprehensive public health response to population ageing. Lancet. 2015;385(9968):658–61. 10.1016/S0140-6736(14)61461-6 WOS:000349214000034. 25468151 PMC4663973

[11] Wang X, Chen P. Population ageing challenges health care in China. Lancet. 2014;383(9920):870–. 10.1016/s0140-6736(14)60443-8 WOS:000332399500024. 24607099

[12] Milner AJ, Carter G, Pirkis J, Robinson J, Spittal MJ. Letters, green cards, telephone calls and postcards: systematic and meta-analytic review of brief contact interventions for reducing self-harm, suicide attempts and suicide. Br J Psychiatry. 2015;206(3):184–90. 10.1192/bjp.bp.114.147819 WOS:000351478900003. 25733570

[13] Wang R, Tang S, Shaw I, Feng Z, Chen Z, Luo Y, et al Integrated decision-making model for community-based rehabilitation service utilisation among persons with severe mental illness in China: protocol for a cross-sectional, mixed-methods study. BMJ Open. 2018;8(12):e021528–e. 10.1136/bmjopen-2018-021528 MEDLINE:30530575. 30530575 PMC6303639

[14] Brown DR, Blanton CJ. Physical activity, sports participation, and suicidal behavior among college students. Med Sci Sports Exerc. 2002;34(7):1087–96. 10.1097/00005768-200207000-00006 WOS:000176710800006. 12131246

[15] Rebar AL, Stanton R, Geard D, Short C, Duncan MJ, Vandelanotte C. A meta-meta-analysis of the effect of physical activity on depression and anxiety in non-clinical adult populations. Health Psychol Rev. 2015;9(3):366–78. 10.1080/17437199.2015.1022901 WOS:000360911000008. 25739893

[16] World Health Organization. Global recommendations on physical activity for health. Geneva: World Health Organization, 2010. 26180873

[17] Elsawy B, Higgins KE. Physical Activity Guidelines for Older Adults. Am Fam Physician. 2010;81(1):55–9. WOS:000273580500008. 20052963

[18] Ku P-W, Fox KR, Chen L-J, Chou P. Physical Activity and Depressive Symptoms in Older Adults 11-Year Follow-Up. Am J Prev Med. 2012;42(4):355–62. 10.1016/j.amepre.2011.11.010 WOS:000301799900005. 22424248

[19] Du W-J, Tan J-P, Yi F, Zou Y-M, Gao Y, Zhao Y-M, et al Physical activity as a protective factor against depressive symptoms in older Chinese veterans in the community: result from a national cross-sectional study. Neuropsychiatr Dis Treat. 2015;11:803–13. 10.2147/NDT.S80295 WOS:000351593600002. 25848278 PMC4376303

[20] Schuch FB, Vancampfort D, Richards J, Rosenbaum S, Ward PB, Stubbs B. Exercise as a treatment for depression: A meta-analysis adjusting for publication bias. J Psychiatr Res. 2016;77:42–51. 10.1016/j.jpsychires.2016.02.023 WOS:000374804700007. 26978184

[21] Joshi S, Mooney SJ, Kennedy GJ, Benjamin EO, Ompad D, Rundle AG, et al Beyond METs: types of physical activity and depression among older adults. Age Ageing. 2016;45(1):103–9. 10.1093/ageing/afv164 WOS:000369089700020. 26764399 PMC4711656

[22] Chi S-H, Wang J-Y, Tsai AC. Combined association of leisure-time physical activity and fruit and vegetable consumption with depressive symptoms in older Taiwanese: Results of a national cohort study. Geriatr Gerontol Int. 2016;16(2):244–51. 10.1111/ggi.12459 WOS:000368809700012. 25657050

[23] Wang S, Ma W, Wang S-M, Yi X. A Cross Sectional Examination of the Relation Between Depression and Frequency of Leisure Time Physical Exercise among the Elderly in Jinan, China. Int J Environ Res Public Health. 2018;15(9). 10.3390/ijerph15092041 MEDLINE:30231530. 30231530 PMC6164447

[24] Kanamori S, Takamiya T, Inoue S, Kai Y, Tsuji T, Kondo K. Frequency and pattern of exercise and depression after two years in older Japanese adults: the JAGES longitudinal study. Sci Rep. 2018;8 10.1038/s41598-018-29053-x WOS:000439686700047. 30046117 PMC6060146

[25] Jung S, Lee S, Lee S, Bae S, Imaoka M, Harada K, et al Relationship between physical activity levels and depressive symptoms in community-dwelling older Japanese adults. Geriatr Gerontol Int. 2018;18(3):421–7. 10.1111/ggi.13195 WOS:000427566500007. 29052928

[26] Heesch KC, Burton NW, Brown WJ. Concurrent and prospective associations between physical activity, walking and mental health in older women. J Epidemiol Community Health. 2011;65(9):807–13. 10.1136/jech.2009.103077 MEDLINE:20515892. 20515892

[27] Buman MP, Hekler EB, Haskell WL, Pruitt L, Conway TL, Cain KL, et al Objective Light-Intensity Physical Activity Associations With Rated Health in Older Adults. Am J Epidemiol. 2010;172(10):1155–65. 10.1093/aje/kwq249 WOS:000283918700008. 20843864 PMC3004766

[28] Bishwajit G, O'Leary DP, Ghosh S, Yaya S, Tang SF, Feng ZC. Physical inactivity and self-reported depression among middle- and older-aged population in South Asia: World health survey. BMC Geriatr. 2017;17:8 10.1186/s12877-016-0394-z WOS:000400891800004. 28454520 PMC5410033

[29] Mammen G, Faulkner G. Physical Activity and the Prevention of Depression A Systematic Review of Prospective Studies. American Journal of Preventive Medicine. 2013;45(5):649–57. 10.1016/j.amepre.2013.08.001 WOS:000325910800016. 24139780

[30] Chang Y-C, Lu M-C, Hu IH, Wu W-CI, Hu SC. Effects of different amounts of exercise on preventing depressive symptoms in community-dwelling older adults: a prospective cohort study in Taiwan. BMJ Open. 2017;7(4). 10.1136/bmjopen-2016-014256 WOS:000402527200073. 28465305 PMC5623457

[31] Wen CP, Wai JPM, Tsai MK, Yang YC, Cheng TYD, Lee MC, et al Minimum amount of physical activity for reduced mortality and extended life expectancy: a prospective cohort study. Lancet. 2011;378(9798):1244–53. 10.1016/S0140-6736(11)60749-6 WOS:000295723600026. 21846575

[32] Vancampfort D, Stubbs B, Firth J, Hallgren M, Schuch F, Lahti J, et al Physical activity correlates among 24,230 people with depression across 46 low- and middle-income countries. J Affect Disord. 2017;221:81–8. 10.1016/j.jad.2017.06.012 WOS:000406464200012. 28633049

[33] Deng Y, Paul DR. The Relationships Between Depressive Symptoms, Functional Health Status, Physical Activity, and the Availability of Recreational Facilities: a Rural-Urban Comparison in Middle-Aged and Older Chinese Adults. Int J Behav Med. 2018;25(3):322–30. 10.1007/s12529-018-9714-3 WOS:000432362600006. 29498014

[34] Olanrewaju O, Kelly S, Cowan A, Brayne C, Lafortune L. Physical Activity in Community Dwelling Older People: A Systematic Review of Reviews of Interventions and Context. PLoS One. 2016;11(12). 10.1371/journal.pone.0168614 WOS:000392842900062. 27997604 PMC5173028

[35] Falck RS, McDonald SM, Beets MW, Brazendale K, Liu-Ambrose T. Measurement of physical activity in older adult interventions: a systematic review. Br J Sports Med. 2016;50(8):464–U87. 10.1136/bjsports-2014-094413 WOS:000373706000007. 26276362

[36] Zhao Y, Hu Y, Smith JP, Strauss J, Yang G. Cohort profile: The China Health and Retirement Longitudinal Study (CHARLS). Int J Epidemiol. 2014;43(1):61–8. 10.1093/ije/dys203 WOS:000332341300012. 23243115 PMC3937970

[37] Boey KW. Cross-validation of a short form of the CES-D in Chinese elderly. Int J Geriatr Psychiatry. 1999;14(8):608–17. 10.1002/(sici)1099-1166(199908)14:8<608::Aid-gps991>3.0.Co;2-z WOS:000082306600002. 10489651

[38] Cheng H, Chen S, McBride O, Phillips MR. Prospective relationship of depressive symptoms, drinking, and tobacco smoking among middle-aged and elderly community-dwelling adults: Results from the China Health and Retirement Longitudinal Study (CHARLS). J Affect Disord. 2016;195:136–43. 10.1016/j.jad.2016.02.023 WOS:000371257400016. 26895091

[39] Cheng ST, Chan ACM. The Center for Epidemiologic Studies Depression Scale in older Chinese: thresholds for long and short forms. Int J Geriatr Psychiatry. 2005;20(5):465–70. 10.1002/gps.1314 WOS:000229247700011. 15852439

[40] Li Y, Chen C, Tu H, Cao W, Fan S, Ma Y, et al Prevalence and risk factors for depression in older people in Xi'an China: a community-based study. Int J Geriatr Psychiatry. 2012;27(1):31–9. 10.1002/gps.2685 WOS:000297795800004. 21284042

[41] Li D, Zhang D-j, Shao J-j, Qi X-d, Tian L. A meta-analysis of the prevalence of depressive symptoms in Chinese older adults. Arch Gerontol Geriatr. 2014;58(1):1–9. 10.1016/j.archger.2013.07.016 WOS:000325984500001. 24001674

[42] Chen R, Copeland JRM, Wei L. A meta-analysis of epidemiological studies in depression of older people in The People's Republic of China. Int J Geriatr Psychiatry. 1999;14(10):821–30. 10.1002/(sici)1099-1166(199910)14:10<821::Aid-gps21>3.0.Co;2-0 WOS:000083239000003. 10521881

[43] Stubbs B, Koyanagi A, Schuch FB, Firth J, Rosenbaum S, Veronese N, et al Physical activity and depression: a large cross-sectional, population-based study across 36 low- and middle-income countries. Acta Psychiatr Scand. 2016;134(6):546–56. 10.1111/acps.12654 WOS:000387852000010. 27704532

[44] Vancampfort D, Stubbs B, Veronese N, Mugisha J, Swinnen N, Koyanagi A. Correlates of physical activity among depressed older people in six low-income and middle-income countries: A community-based cross-sectional study. Int J Geriatr Psychiatry. 2018;33(2):E314–E22. 10.1002/gps.4796 WOS:000422754300028. 28994143

[45] Mc Dowell CP, Carlin A, Capranica L, Dillon C, Harrington JM, Lakerveld J, et al Associations of self-reported physical activity and depression in 10,000 Irish adults across harmonised datasets: a DEDIPAC-study. BMC Public Health. 2018;18 10.1186/s12889-018-5702-4 WOS:000437492000001. 29960595 PMC6026508

[46] Lei X, Sun X, Strauss J, Zhang P, Zhao Y. Depressive symptoms and SES among the mid-aged and elderly in China: Evidence from the China Health and Retirement Longitudinal Study national baseline. Soc Sci Med. 2014;120:224–32. 10.1016/j.socscimed.2014.09.028 WOS:000345180600026. 25261616 PMC4337774

[47] Bai X, Lai DWL, Guo A. Ageism and Depression: Perceptions of Older People as a Burden in China. J Soc Iss. 2016;72(1):26–46. 10.1111/josi.12154 WOS:000372524100002.

[48] Zhu W, Chi A, Sun Y. Physical activity among older Chinese adults living in urban and rural areas: A review. Journal of Sport and Health Science. 2016;5(3):281–6. 10.1016/j.jshs.2016.07.004 WOS:000388415100005. 30356525 PMC6188614

[49] Muntner P, Gu DF, Wildman RP, Chen JC, Qan WQ, Whelton PK, et al Prevalence of physical activity among Chinese adults: Results from the International Collaborative Study of Cardiovascular Disease in Asia. Am J Public Health. 2005;95(9):1631–6. 10.2105/AJPH.2004.044743 WOS:000231548700027. 16051938 PMC1449408

[50] Ng SW, Howard AG, Wang HJ, Su C, Zhang B. The physical activity transition among adults in China: 1991–2011. Obes Rev. 2014;15:27–36. 10.1111/obr.12127 WOS:000334764900005. 24341756 PMC3869092

[51] Wang CW, Chan CLW, Yip PSF. Suicide rates in China from 2002 to 2011: an update. Soc Psychiatry Psychiatr Epidemiol. 2014;49(6):929–41. 10.1007/s00127-013-0789-5 WOS:000336287900009. 24240568

[52] Zhong BL, Chiu HFK, Conwell Y. Elderly suicide trends in the context of transforming China, 1987–2014. Sci Rep. 2016;6:9 10.1038/s41598-016-0002-7 WOS:000388557900001. 27886219 PMC5123573

[53] Mutrie N, Hannah MK. The importance of both setting and intensity of physical activity in relation to non-clinical anxiety and depression International Journal of Health Promotion and Education 2007;45(1):24–32.

[54] Andrade-Gomez E, Martinez-Gomez D, Rodriguez-Artalejo F, Garcia-Esquinas E. Sedentary behaviors, physical activity, and changes in depression and psychological distress symptoms in older adults. Depression and Anxiety. 2018;35(9):884–97. 10.1002/da.22804 WOS:000443554000009. 30040170

[55] Wang F, DesMeules M, Luo W, Dai S, Lagace C, Morrison H. Leisure-Time Physical Activity and Marital Status in Relation to Depression Between Men and Women: A Prospective Study. Health Psychol. 2011;30(2):204–11. 10.1037/a0022434 WOS:000288418200011. 21401254

[56] Fassberg MM, van Orden KA, Duberstein P, Erlangsen A, Lapierre S, Bodner E, et al A Systematic Review of Social Factors and Suicidal Behavior in Older Adulthood. Int J Environ Res Public Health. 2012;9(3):722–45. 10.3390/ijerph9030722 WOS:000302175400006. 22690159 PMC3367273

[57] Litwin H. Physical activity, social network type, and depressive symptoms in late life: An analysis of data from the National Social Life, Health and Aging Project. Aging Ment Health. 2012;16(5):608–16. 10.1080/13607863.2011.644264 WOS:000305977500008. 22296412 PMC3430832

[58] Lawlor DA, Taylor M, Bedford C, Ebrahim S. Is housework good for health? Levels of physical activity and factors associated with activity in elderly women. Results from the British Women's Heart and Health Study. J Epidemiol Community Health. 2002;56(6):473–8. 10.1136/jech.56.6.473 WOS:000175768100021. 12011209 PMC1732184

[59] Rothon C, Edwards P, Bhui K, Viner RM, Taylor S, Stansfeld SA. Physical activity and depressive symptoms in adolescents: a prospective study. BMC Med. 2010;8 10.1186/1741-7015-8-32 WOS:000279917100001. 20509868 PMC2895574

